# Postprandial Hyperlipidemia: Association with Inflammation and Subclinical Atherosclerosis in Patients with Rheumatoid Arthritis

**DOI:** 10.3390/biomedicines10010133

**Published:** 2022-01-08

**Authors:** Natalia Mena-Vázquez, Rocío Redondo-Rodríguez, José Rioja, Francisco Gabriel Jimenez-Nuñez, Sara Manrique-Arija, Jose Manuel Lisbona-Montañez, Laura Cano-García, Marta Rojas-Gimenez, Inmaculada Ureña, Pedro Valdivielso, Antonio Fernández-Nebro

**Affiliations:** 1 Instituto de Investigación Biomédica de Málaga (IBIMA), 29010 Malaga, Spain; rocio.redondo.sspa@juntadeandalucia.es (R.R.-R.); jose.rioja@uma.es (J.R.); franciscop.jimenez.sspa@juntadeandalucia.es (F.G.J.-N.); sara.manrique.sspa@juntadeandalucia.es (S.M.-A.); laura.cano.sspa@juntadeandalucia.es (L.C.-G.); inmaculada.urena.sspa@juntadeandalucia.es (I.U.); valdivielso@uma.es (P.V.); afernandezn@uma.es (A.F.-N.); 2 UGC de Reumatología, Hospital Regional Universitario de Málaga, 29009 Malaga, Spain; josemanuellisbona@uma.es; 3 Departamento de Medicina y Dermatología, Universidad de Málaga, 29010 Malaga, Spain; 4 UGC de Reumatología, Instituto Maimónides de Investigación Biomédica de Cordoba (IMIBIC), Hospital Universitario Reina Sofia, 14004 Cordoba, Spain; marta.rojas.gimenez.sspa@juntadeandalucia.es; 5 UGC de Medicina Interna, Hospital Universitario Virgen de la Victoria, 29010 Malaga, Spain

**Keywords:** rheumatoid arthritis, postprandial lipemia, triglycerides, apolipoprotein B48, inflammation, subclinical atherosclerosis

## Abstract

Objective: To describe postprandial lipidemia in patients with rheumatoid arthritis (RA) and to analyze its association with subclinical atherosclerosis and inflammatory activity. Methods: Observational study of 80 cases of RA and 80 sex- and age-matched controls. We excluded individuals with dyslipidemia. Postprandial hyperlipidemia (PPHL) was defined as postprandial triglycerides >220 mg/dL and/or postprandial ApoB48 levels >75th percentile (>p75). Plasma lipids, cholesterol, triglycerides, ApoB48, and total ApoB were evaluated at baseline and after a meal. Other variables analyzed included subclinical atherosclerosis (defined as presence of carotid atheromatous plaque), inflammatory activity (disease activity score (DAS28-ESR)), cytokines, apolipoproteins, and physical activity. A multivariate analysis was performed to identify factors associated with PPHL in patients with RA. Results: A total of 75 patients with RA and 67 healthy controls fulfilled the inclusion criteria. PPHL was more frequent in patients with RA than controls (No. (%), 29 (38.70) vs. 15 (22.40); *p* = 0.036), as was subclinical atherosclerosis (No. (%), 22 (30.10) vs. 10 (14.90); *p* = 0.032). PPHL in patients with RA was associated with subclinical atherosclerosis (OR (95% CI) 4.69 (1.09–12.11); *p* = 0.037), TNF-α (OR (95% CI) 2.00 (1.00–3.98); *p* = 0.048), high-sensitivity C-reactive protein (OR (95% CI) 1.10 (1.01–1.19); *p* = 0.027), and baseline triglycerides (OR (95% CI) 1.02 (1.00–1.04); *p* = 0.049). Conclusion: PPHL was more frequent in patients with RA than in controls. PPHL in patients with RA was associated with inflammation and subclinical atherosclerosis.

## 1. Introduction

Rheumatoid arthritis (RA) is a chronic inflammatory disease characterized by persistent sinusitis, bone erosion, and functional incapacity. RA is associated with premature death and multiple morbidities [1], mainly because the associated cardiovascular risk is similar to that of patients with type 2 diabetes mellitus [2]. This increased risk can be explained by the presence of traditional and nontraditional cardiovascular risk factors, including systemic inflammation and dyslipidemia [3,4,5,6,7].

Interest in triglycerides as a cardiovascular risk factor is growing, since elevated postprandial lipidemia was recently shown to be an independent predictor of the risk of arteriosclerosis in the general population [8,9]. This triglyceride intolerance syndrome results from difficulty processing triglyceride-rich lipoproteins, which in turn increase the risk of atherosclerosis [10,11]. Recent studies on postprandial lipidemia evaluated ApoB48 levels 4 h after a mixed breakfast, since this correlates well with the area under the curve for triglycerides after 8 h [12,13] and reflects the number of circulating chylomicrons and their remnants. Furthermore, postprandial triglyceride levels >220 mg/dL are also considered abnormally high [14]. Postprandial lipidemia has been shown to be higher in RA patients than healthy controls, and an association between postprandial ApoB48 levels and pathologic carotid intima media thickness (cIMT) has been reported [15,16].

Therefore, considering that for most of the day we are in the postprandial state, the impact of postprandial hyperlipidemia (PPHL) on cardiovascular risk in patients with RA seems to be much greater than expected. However, few studies have examined factors associated with hyperlipidemia under fasting and postprandial conditions. In diabetic patients with coronary disease and peripheral artery disease, ApoB48 is positively associated with triglycerides and body mass index (BMI) and negatively associated with high-density lipoproteins (HDL) [17,18]. In RA, higher ApoB48 values are associated with positive titers of anticyclic citrullinated peptide antibody (ACPA) and rheumatoid factor (RF). In contrast, no association was found between inflammatory activity and the use of disease-modifying drugs, possibly because other inflammatory parameters were not assessed and baseline ApoB48 was measured instead of postprandial ApoB48 [16]. No factors associated with postprandial hyperlipidemia have been identified in patients with RA. Some inflammatory cytokines involved in the pathogenesis of RA, have been shown to significantly increase in triglyceride levels such as TNF-α [19]. In this way, there could be an association between increase in postprandial lipidemia and inflammation in RA patients.

In a previous study [15], we observed that patients with RA experienced a more marked increase in postprandial lipidemia than healthy controls and that ApoB48 levels were associated with cIMT. However, the factors associated with this increase in ApoB48 were not analyzed because they were not the objective of the study. Therefore, the objectives of the present study were as follows: (1) to study clinical-laboratory factors and activity and severity of RA associated with postprandial lipidemia; and (2) to confirm the association between postprandial lipidemia and subclinical atherosclerosis in a larger sample.

## 2. Materials and Methods

### 2.1. Design

A cross-sectional study of a cohort of patients with established RA and healthy sub-jects was carried out at Hospital Regional Universitario de Málaga and the Lipid and Ar-teriosclerosis Laboratory of the University of Malaga in Spain. Protocol was approved by the Clinical Research Ethics Committee of HRUM on 16 November 2018 (Code 1787-N-18). All of the patients gave their written informed consent to participate.

### 2.2. Patients

All patients were consecutively recruited between January 2019 and February 2021 from a prospective inception cohort of RA patients. The selection criteria were: diagnosis of RA according to the 2010 criteria of American College of Rheumatology/European League Against Rheumatism [20], age of onset greater than 16 years old, and uninterrupted prospective follow-up to the cut-off date. Patients with inflammatory diseases other than RA (except secondary Sjögren syndrome), patients with dyslipidemia or treated with lipid-lowering therapy, patients with active infection, and pregnant women were excluded.

### 2.3. Controls

The control group comprised volunteers aged >16 years who had been randomly selected from a health center in the catchment area. They did not have inflammatory or autoimmune diseases or symptoms that might lead these diseases to be suspected. The exclusion criteria for controls were the same as for patients. Controls and patients were matched by sex and age.

### 2.4. Protocol

Fasting blood samples were drawn early in the morning. All participants, including patients and control group, had a mixed breakfast in the hospital (milk, ham, cheese, oil, and bread). The nutrient values were as follows: 775 kcal; fat, 50 g (53% saturated fatty acids, 41% monounsaturated fatty acids, 6% polyunsaturated fatty acids); and carbohydrates, 40 g. During the postprandial period, participants had to rest and not smoke. Four hours later, a second blood sample was drawn. Samples to be used for lipid analysis were frozen for further processing. On the same day, all participants were clinically assessed by a rheumatologist following a pre-established protocol, and a carotid ultrasound scan was performed.

### 2.5. Dependent Variable

The dependent variable was PPHL, which was defined as postprandial triglycerides >220 mg/dL and/or postprandial ApoB48 levels above the 75th percentile (>p75) [14]. Postprandial ApoB48 levels above the 75th percentile (>p75) was calculated according to the results of the present study. These were determined in plasma samples after fasting and 4 h after a meal using enzymatic techniques (SPINREACT, Barcelona, Spain) in a Mindray BS 380 autoanalyzer (MINDRAY, Shenzhen, China) to triglyceride. Currently there are no commercial quality controls available for apoB48, for this reason we have used a local internal quality control was pooled according to WHO recommendations and assayed by duplicate on each plate in parallel to samples. Calculated variation coefficients were 12.4% (total), 11.0% (inter-assay), and 5.7% (intra-assay) [21].

### 2.6. Other Recorded Variables

Other variables included expected factors associated with postprandial hyperlipidemia, such as inflammatory activity in arthritis, severity, apolipoproteins, and subclinical atherosclerosis.

Inflammatory activity was evaluated using the 28-joint disease activity score with erythrocyte sedimentation rate (DAS28-ESR) (continuous, range 0–9.4) [22], which was recorded at the visit [23]. According to DAS28-ESR, high activity was defined as >5.1, moderate as 3.2–5.1, low as 2.6–3.2, and remission as ≤2.6. We also evaluated inflammatory activity based on a Cytokine 25-Plex Human ProcartaPlex™ Panel 1B (Thermofisher Scientific, Madrid, Spain), including the following cytokines: GM-CSF; IFN alpha; IFN gamma; IL-1α; IL-1ß; IL-1RA; IL-2; IL-4; IL-5; IL-6; IL-7; IL-9; IL-10; IL-12 p70; IL-13; IL-15; IL-17A; IL-18; IL-21; IL-22; IL-23; IL-27; IL-31; TNF-α; TNF-ß. Data acquisition was performed in a Bio-Plex 200 reader (Bio-Rad, Hercules, CA, USA). Serum levels of high-sensitivity C-reactive protein (hsCRP) were determined using turbidimetry (BIOSYSTEMS, Barcelona, Spain). Severity was assessed based on antibodies and their titers (RF positive if >10 IU/mL; ACPA positive if >20 IU/mL), the presence of erosions in imaging, and the Health Assessment Questionnaire (HAQ) score [23].

The laboratory variables included serum lipids, cholesterol, and triglycerides [24], which were measured using enzyme-based techniques (SPINREACT, Barcelona, Spain) in a Mindray BS 380 automatic chemistry analyzer. Cholesterol content and triglycerides in chylomicrons and very-low-density lipoproteins were measured using sequential ultracentrifugation [25] with the same enzyme-based kits. HDL cholesterol was measured using a homogeneous assay (SIEMENS, Germany). Low-density lipoprotein (LDL) cholesterol was calculated using the Friedewald formula.

We also analyzed the role of apolipoprotein A1 (Apo A1), which is the main protein component of HDL and expresses its antiatherogenic properties by transporting cholesterol to liver tissue [16]. Subclinical atherosclerosis was defined as the presence of atheromatous plaque defined as focal thickening of the arterial wall projecting toward the lumen and measuring >0.5 mm or more than 50% of the neighboring cIMT value or cIMT >1.5 mm [26]. Pathologic cIMT was defined as a carotid thickness greater than the 90th percentile (>p90) for age and sex according to data for the Spanish population [27]. Carotid ultrasound was performed using the ART.LAB system (Esaote) by a trained rheumatologist with experience in the technique.

### 2.7. Remaining Variables

We recorded a series of epidemiological variables at the cut-off, as follows: sex, race, BMI (weight/height in m^2^), waist circumference (cm), hip circumference (cm), waist–hip ratio (waist circumference/hip circumference, cm), comorbid conditions related to traditional cardiovascular risk factors (smoking (active, former, never), obesity (BMI > 30), arterial hypertension (arterial blood pressure ≥ 140/90 mmHg), or current antihypertensive therapy) [28], diabetes diagnosed according to the criteria of the American Diabetes Association [29], and a personal history of cardiovascular disease. Family history of coronary disease was defined as a first-degree relative with myocardial infarction or stroke <55 years in men and <60 years in women. We applied the following instruments: a validated survey on adherence to the Mediterranean diet using the Mediterranean Diet Adherence Screener (MEDAS) [30], a 14-item questionnaire according to which the patient is considered to be adhering if the score is ≥9 and not adhering if the score is <9; the International Physical Activity Questionnaire (IPAQ) [31], expressed in metabolic equivalent of task (MET) units (low/sedentary physical activity or insufficient to fulfill the recommendations for healthy activity (<600 MET-minute during the previous week) or moderate/high or fulfilling the recommendations at a moderate level (>600 MET-minutes in the previous week)). We also recorded treatment with conventional synthetic disease-modifying drugs (csDMARDs) and biologic DMARDs (bDMARDs).

### 2.8. Statistical Analysis

We performed a descriptive analysis of epidemiological and clinical characteristics, as well as of the lipid profile and carotid ultrasound findings, of both patients and controls. The normal quantitative variables were expressed as mean and standard deviation (SD), and the skewed variables as a median and interquartile range (IQR). Qualitative variables were expressed as absolute numbers. Normality of the distribution, as assessed using the Kolmogorov–Smirnov test. The χ^2^ and *t* test or Mann–Whitney test were used to compare the main characteristics between patients and controls, as well as between patients with RA and PPHL and patients with RA but not PPHL. The Wilcoxon test was used to compare mean ranges between variables associated with baseline and postprandial lipid values. Finally, we constructed 3 logistic regression models to identify factors associated with PPHL in patients with RA, healthy group and total sample. Accepting an α risk of 0.05 and a ß risk of 0.2 in a 2-sided contrast, the sample size required was 78 individuals in each group to detect characteristics of RA associated with postprandial lipidemia, such as the disease itself [16]. The sample size was calculated assuming that 10% of patients may be finally excluded from analysis for not meeting inclusion criteria. Statistical significance was set at *p* < 0.05. The analysis was performed using R 2.4-0.

## 3. Results

**Figure 1:**
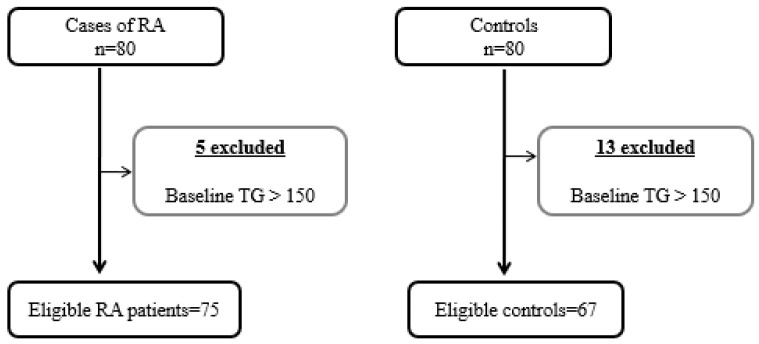
Flowchart showing patients included in the study.

The initial study population comprised 80 patients and 80 controls; however, only 75 patients with RA (93.80%) and 67 healthy controls (83.80%) fulfilled the selection criteria. Figure 1 shows the progress of participants through the study.

### 3.1. Characteristics of the Study Population

Table 1 shows the baseline characteristics of patients and controls. Both groups were evenly balanced. However, in the patients’ group, there were more smokers, acute phase reactant and antibody values were higher, and participants did less physical exercise.

Most patients were women with established RA (mean (SD) duration of disease, 135.22 (68.90) months); a total of 34/75 patients (46.00%) had erosive disease, and more than half were in remission or had low disease activity. All of the patients were receiving a DMARD (csDMARD in 66/75 (88.00%), bDMARD in 36/75 (48.00%), mainly anti-TNF- agents). A total of 24/75 patients (32.00%) were receiving corticosteroids.

As shown in Table 2, more patients than controls had pathologic cIMT (36.00% vs. 19.40%; *p* = 0.028) and atheromatous plaque (30.10% vs. 14.9%; *p* = 0.032). No significant differences were found in the number of atheromatous plaques or in 2-sided involvement. As for proinflammatory cytokines, RA patients generally had higher values than controls for GM-CSF, IL-1ß, IL-12, IL-13, IL-2, IL-5, IL-6, TNF-α, IL-10, IL-17A, IL-21, IL-22, IL-23, IL-27, IL-9, IL1α, IL-15, IL-1RA, IL-7, and TNF-ß.

### 3.2. Pre- and Postprandial Lipidemia in Patients and Controls

Table 3 shows baseline and postprandial data for lipid profile in patients and controls. While both groups had very similar lipid parameters, more patients with RA had PPHL than controls (No. (%) = 29 (38.70) vs. 15 (22.40); *p* = 0.036), albeit with a marked increase in postprandial ApoB48 levels (mean (SD) = 6.82 (4.55) vs. 5.39 (3.86); *p* = 0.043).

### 3.3. Factors Associated with Postprandial Hyperlipidemia in Patients with Rheumatoid Arthritis

As seen in Table 4, of the 75 patients with RA, 29 had PPHL (38.7%). These patients were characterized by more frequent smoking (*p* = 0.048) and obesity (*p* = 0.014) and a higher waist-hip ratio (*p* = 0.007). Furthermore, patients with PPHL more frequently had positive ACPA titers (*p* = 0.043) and greater mean DAS28-ESR scores (*p* = 0.042) and hsCRP levels (*p* = 0.040). There were no differences between the groups for csDMARDs, bDMARDs, or corticosteroids.

Furthermore, cIMT values were higher in patients with RA and PPHL (*p* = 0.045), as was the frequency of pathologic cIMT (*p* = 0.003), atheromatous plaque (*p* = 0.040), and 2-sided involvement (*p* = 0.018). As for proinflammatory cytokines, patients with RA and PPHL had higher median (IQR) levels of IL-18 (21.65 (10.80–26.75) vs. 13.29 (10.17–19.23); *p* = 0.039) and TNF-α (1.49 (0.98–5.16) vs. 1.14 (0.98–1.68); *p* = 0.036) than the other patients. No differences were observed for the remaining cytokines.

### 3.4. Multivariate Analysis

The factors associated with PPHL in the total sample (cases and controls) were RA diagnosis, baseline TG levels and obesity (Table 5), while in the healthy control group, only baseline TG levels (OR (95% CI) 1.02 (1.00–1.03); *p* = 0.012) and obesity (BMI ≥ 30) (OR (95% CI) 2.13 (1.00–4.29); *p* = 0.045) (R2 = 0.221) were identified. Table 6 shows the results of the multivariate logistic regression analysis for the dependent variable PPHL in patients with RA. The presence of carotid atheromatous plaque and high levels of TNF-α, hsCRP, and triglycerides at baseline was independently associated with PPHL in patients with RA. These factors explain 51% of the variability in the presence of PPHL in these patients (R2 = 0.516). Other multivariate models that included biological treatment and anti TNF-α treatment had no influence on the results.

## 4. Discussion

We evaluated postprandial lipidemia in RA patients and controls to describe the potential association with subclinical arteriosclerosis based on measurement of atheromatous plaque. We also evaluated whether PPHL was associated with other clinical and laboratory factors, inflammatory activity, and severity of RA.

This approach showed that patients with RA had fasting triglyceride and ApoB48 values that were similar to those of controls, although PPHL was more frequent in patients with RA (*p* = 0.036). In this sense, the diagnosis of RA was one of the factors independently associated with PPHL in the total sample (cases and controls). We defined PPHL as postprandial triglycerides >220 mg/dL and/or postprandial ApoB48 levels greater than the 75th percentile (>p75) [14]. The highest postprandial ApoB48 values reported to date in patients with RA were those previously published by our group [15]. Burggraaf et al. [16] also reported that patients with RA had higher baseline ApoB48 levels than controls and that the accumulation of atherogenic chylomicron remnants could contribute to the risk of cardiovascular events in affected patients. In addition, while triglyceride values >220 mg/dL were more common in patients with RA in our study, the difference was not statistically significant. Similarly, Burggraaf et al. [16] found no differences in triglyceride levels between patients and controls, suggesting that lipolysis of chylomicrons may be at least altered in patients with RA and that, perhaps, abnormal catabolism of chylomicron remnants in the liver may be predominant.

As for subclinical atherosclerosis, more patients than controls had atheromatous plaque and pathologic cIMT. This finding has been reported elsewhere [32,33,34,35]. In fact, a recent systematic review and meta-analysis showed that patients with RA had a significantly higher cIMT than the control group and that this could be affected by age, BMI, disease duration, and inflammatory activity [32]. Similarly, the presence of atheromatous plaques was associated with PPHL in patients with RA. Previous studies have associated postprandial ApoB48 with pathologic cIMT [11,15,36]. Chylomicron remnants have a direct effect on the development of atherosclerosis, since their lower size enables them to reach the subendothelial region and trigger inflammation and atherosclerosis [37], whereas chylomicrons can exert an indirect effect, by stimulating inflammation via activation of circulating leukocytes and formation of foam cells [21,38,39,40]. Thus, PPHL is becoming increasingly interesting, given that postprandial triglycerides can predict cardiovascular events in much the same way as—or even better than—fasting triglycerides, with the practical advantage that the patient does not need to fast [8,40,41]. Similarly, baseline triglyceride levels have also been associated with PPHL in patients with RA. Other study [37] showed that baseline triglycerides, chylomicrons, and chylomicron remnants can activate leukocytes and that both activation of leukocytes and PPHL have been associated with the presence and development of atherosclerosis.

We also found hsCRP and TNF-α to be associated with PPHL in patients with RA. No studies to date have evaluated the role of these factors in PPHL in patients with RA. However, after adjusting for various confounders, Jeong et al. [42] found that hsCRP levels were associated with a greater prevalence of hypertriglyceridemia, diabetes, and metabolic syndrome. Similarly, a study of patients with RA whose disease was considered to be clinically controlled according to activity indices, hsCRP levels were associated with increased cardiovascular risk [43]. The proinflammatory cytokine TNF-α is involved in the pathogenesis of RA and has been shown to have effects on the metabolism of lipoproteins, namely, that it diminishes lipoprotein lipase activity and hepatic metabolism [44]. A systematic review also revealed that TNF-α values were associated with a significant increase in triglyceride levels [19].

The main strength of the present study is that it is the first to evaluate factors associated with PPLH in patients with RA. However, the study is also subject to a series of limitations. First, it was cross-sectional in design and describes the association between PPHL in patients with RA and inflammation-related factors and subclinical atherosclerosis. Longitudinal studies would be necessary to determine whether subclinical atherosclerosis could lead to cardiovascular events. Second, while other studies have evaluated PPHL as an increase in the area under the curve for triglycerides after an oral fat load [45], we measured postprandial lipidemia as a composite variable taking into account postprandial ApoB48 and triglyceride levels. Nevertheless, we believe that our findings are reliable, since other studies have already shown that determination of ApoB48 and triglycerides >200 mg/dL at 4 h after a mixed breakfast correlates well with the area under the curve for triglycerides after 8 h [12]. The odds ratio values in this study are close to 1 and the higher values also have wider confidence intervals. Probably subsequent studies with a larger sample size are needed to have results with greater power. On the other hand, 5 patients with RA and 13 healthy controls did not meet the selection criteria by baseline TG > 150. There was a loss of 2% greater than that estimated in the sample calculation, however this deviation was not considered relevant. Nonetheless, statistically significant differences are observed in the study objectives. Finally, the evaluation of LDL levels by derivation from the Friedewald’s formula may not be entirely accurate with triglyceride levels >200 mg/dL. However, the deviation is small (LDL is 18.4 mg/dL higher if triglyceride levels are between 200–399) according to some studies [46] and the postprandial hypertriglyceridemia values observed in our study were moderate. Likewise, there seems to be a good correlation between Friedewald’s formula and the enzymatic method in patients with triglyceride levels below 400 [47].

## 5. Conclusions

In conclusion, postprandial hyperlipidemia values are higher in patients with RA than in controls, even though fasting triglycerides were similar in both groups. Our results also show that atheromatous plaques, TNF-α, hsCRP, and fasting triglycerides are all associated with PPHL in patients with RA. We demonstrate a clear association between PPHL and inflammation and subclinical atherosclerosis in patients with RA.

## Figures and Tables

**Table 1:** Clinical–analytical characteristics of 75 patients with RA and 67 controls.

Variable	Patients *n* = 75	Controls *n* = 67	*p* Value
Epidemiologic characteristics			
Age in years, mean (SD)	54.87 (11.45)	53.58 (11.38)	0.600
Female sex; *n* (%)	64 (85.30)	59 (88.10)	0.634
Smoking			0.045
Never smoked, *n* (%)	34 (45.30)	44 (65.70)	
Ex-smoker, *n* (%)	26 (34.70)	13 (19.40)	
Smoker, *n* (%)	15 (20.00)	10 (14.90)	
Comorbidities			
Arterial hypertension, *n* (%)	21 (28.00)	12 (17.90)	0.155
Diabetes mellitus, *n* (%)	3 (4.00)	4 (6.00)	0.588
Cardiovascular disease, *n* (%)	5 (6.70)	1 (1.50)	0.126
Family history of coronary disease, *n* (%)	21 (28.00)	10 (14.90)	0.060
Anthropometric characteristics			
BMI (kg/m^2^), mean (SD)	27.65 (5.24)	26.82 (4.95)	0.326
Obesity (BMI ≥ 30), *n* (%)	15 (22.70)	17 (22.70)	0.972
Waist circumference, (cm), median (IQR)	90.00 (83.00–102.00)	89.50 (80.00–100.00)	0.363
Hip circumference (cm), median (IQR)	105.50 (99.75–110-00)	104.50 (97.75–110.00)	0.573
Waist-hip ratio, median (IQR)	0.87 (0.81–0.91)	0.86 (0.81–0.90)	0.658
MET-minutes, median (IQR)	460.00 (215.00–672.20)	693.00 (396.00–1020.00)	0.005
Total MEDAS, median (IQR)	9.00 (8.00–10.00)	9.00 (8.00–11.00)	0.890
Clinical-laboratory characteristics			
Time since diagnosis of RA, months, mean (SD)	135.22 (68.90)	-	-
Diagnostic delay, months, median (IQR)	5.74 (5.62–9.76)	-	-
Erosions, *n* (%)	34 (45.90)	-	-
RF > 10, *n* (%)	62 (82.70)	0 (0.00)	<0.001
ACPA > 20, *n* (%)	59 (78.70)	0 (0.00)	<0.001
High-sensitivity CRP (mg/dL), median (IQR)	4.72 (2.50–7.87)	1.76 (0.80–2.98)	<0.001
ESR (mm/h), median (IQR)	16.00 (8.00–25.00)	11.00 (6.00–18.00)	0.008
NPJ (0–28), median (IQR)	1.00 (0.00–2.00)	-	-
NSJ (0–28), median (IQR)	0.00 (0.00–1.00)	-	-
DAS28-ESR at cut-off, median (IQR)	2.76 (2.22–4.11)	-	-
Remission-low activity, *n* (%)	45 (60.00)	-	-
Moderate-high activity, *n* (%)	30 (40.00)	-	-
HAQ, median (IQR)	0.85 (0.25–1.75)	-	-
Treatments			
Synthetic DMARDs, *n* (%)	66 (88.00)	-	-
Methotrexate, *n* (%)	46 (61.30)	-	-
Leflunomide, *n* (%)	7 (9.30)	-	-
Sulfasalazine, *n* (%)	6 (8.00)	-	-
Hydroxychloroquine, *n* (%)	6 (8.00)		
Biologic DMARDs, *n* (%)	36 (48.00)	-	-
Anti TNF-α, *n* (%)	23 (30.70)	-	-
Jak inhibitor, *n* (%)	3 (4.00)	-	-
Anti-IL-6, *n* (%)	7 (9.30)	-	-
Abatacept, *n* (%)	1 (1.30)		
Rituximab, *n* (%)	2 (2.70)		
Corticosteroids at cut-off, *n* (%)	24 (32.00)	-	-
Dose of corticosteroids at cut-off, median (IQR)	5.00 (5.00–10.00)	-	-

Abbreviation: BMI: body mass index; ACPA: anticyclic citrullinated peptide antibody; RF: rheumatoid factor. SD: standard deviation, MEDAS: Mediterranean Diet Adherence Screener (validated questionnaire); DAS28-ESR (28-joint disease activity score); NPJ: number of painful joints; NSJ, number of swollen joints; HAQ: Health Assessment Questionnaire; CRP: C-reactive protein; ESR: erythrocyte sedimentation rate; DMARD, disease-modifying antirheumatic drug; Il-6: interleukin 6; Anti-TNF: anti–tumor necrosis factor.

**Table 2:** cIMT study and inflammatory cytokine profile for 75 patients with RA and 67 controls.

Variable	RA Patients (*n* = 75)	Controls (*n* = 67)	*p* Value
Carotid ultrasound			
Pathologic cIMT > p90, mean (SD)	27 (36.00)	13 (19.40)	0.028
cIMT (mm), mean (SD)	0.75 (0.13)	0.73 (0.12)	0.507
Right cIMT (mm), median (IQR)	0.68 (0.58–0.78)	0.66 (0.61–0.70)	0.706
Left cIMT (mm), mean (SD)	0.71 (0.12)	0.70 (0.12)	0.545
Patients with atheromatous plaque, *n* (%)	22 (30.10)	10 (14.90)	0.032
Two-sided involvement, *n* (%)	6 (8.20)	5 (7.50)	0.868
≥2 plaques, *n* (%)	8 (11.00)	5 (7.50)	0.476
Cytokines			
GM-CSF, pg/mL, median (IQR)	8.21 (7.91–10.39)	7.91 (7.20–8.54)	0.002
IFN-γ, pg/mL, median (IQR)	1.28 (0.99–2.14)	1.51 (1.07–2.23)	0.410
IL-1ß, pg/mL, median (IQR)	0.40 (0.32–0.50)	0.32 (0.30–0.40)	<0.001
IL-12 p70, pg/mL, median (IQR)	0.15 (0.09–0.23)	0.11 (0.09–0.13)	<0.001
IL-13, pg/mL, median (IQR)	2.10 (1.77–2.86)	1.98 (1.56–2.19)	0.001
IL-18, pg/mL, median (IQR)	15.39 (10.61–25.07)	12.87 (9.18–18.87)	0.143
IL-2, pg/mL, median (IQR)	2.23 (2.04–2.42)	2.04 (1.66–2.04)	<0.001
IL-4, pg/mL, median (IQR)	2.24 (1.92–2.97)	2.24 (1.72–2.61)	0.054
IL-5, pg/mL, median (IQR)	2.43 (2.21–2.87)	2.21 (1.78–2.65)	0.001
IL-6, pg/mL, median (IQR)	0.78 (0.56–1.70)	0.70 (0.56–0.86)	0.006
TNF-α, pg/mL, median (IQR)	1.23 (0.98–2.25)	0.98 (0.90–1.14)	<0.001
IL-10, pg/mL, median (IQR)	0.19 (0.13–0.39)	0.13 (0.10–0.16)	<0.001
IL-17A, pg/mL, median (IQR)	0.90 (0.59–2.58)	0.59 (0.48–0.65)	<0.001
IL-21, pg/mL, median (IQR)	10.09 (2.21–72.82)	1.60 (0.93–6.48)	<0.001
IL-22, pg/mL, median (IQR)	11.39 (3.12–138.80)	3.10 (1.76–9.50)	<0.001
IL-23, pg/mL, median (IQR)	0.42 (0.40–0.52)	0.40 (0.29–0.40)	<0.001
IL-27, pg/mL, median (IQR)	6.28 (4.93–7.64)	6.28 (4.93–6.28)	0.005
IL-9, pg/mL, median (IQR)	0.13 (0.10–0.20)	0.13 (0.09–0.14)	<0.001
IFN-α, pg/mL, median (IQR)	0.10 (0.08–0.11)	0.08 (0.07–0.09)	0.005
IL-1α, pg/mL, median (IQR)	2.41 (0.45–18.94)	0.2 (0.09–0.94)	<0.001
IL-15, pg/mL, median (IQR)	2.91 (2.71–3.54)	2.48 (2.40–2.70)	<0.001
IL-1RA, pg/mL, median (IQR)	92.84 (61.76–307.60)	67.53 (43.73–103.31)	<0.001
IL-31, pg/mL, median (IQR)	0.91 (0.78–1.06)	0.91 (0.78–0.91)	0.510
IL-7, pg/mL, median (IQR)	1.17 (0.70–1.67)	0.87 (0.55–1.59)	0.023
TNF-ß, pg/mL, median (IQR)	0.11 (0.10–0.14)	0.10 (0.09–0.11)	0.002

Abbreviations; cIMT: carotid intima-media thickness; SD: standard deviation; IQR: interquartile range; GM-CSF: granulocyte macrophage-colony stimulating factor; IFN: interferon; IL: interleukin; TNF: tumor necrosis factor.

**Table 3:** Lipid profile for 75 patients with RA and 67 controls.

Variable	Patients *n* = 75	Controls *n* = 67	*p* Value
Preprandial lipid profile			
Total cholesterol (mg/dL), mean (SD)	205.60 (35.25)	199.85 (34.86)	0.328
LDL cholesterol (mg/dL), mean (SD)	122.97 (30.49)	119.14 (29.68)	0.451
HDL cholesterol (mg/dL), mean (SD)	62.62 (18.37)	66.21 (23.90)	0.322
Triglycerides (mg/dL), median (IQR)	84.00 (67.05–111.00)	81.10 (64.00–110.00)	0.955
ChMTG, median (IQR)	14.73 (8.42–25.40)	15.20 (7.92–28.21)	0.761
ChMchol, median (IQR)	10.18 (7.59–13.63)	8.99 (5.62–14.91)	0.291
VLDLTG, median (IQR)	13.43 (8.72–20.31)	10.99 (7.20–19.49)	0.218
VLDLchol, median (IQR)	7.41 (3.56–11.42)	7.39 (4.22–11.00)	0.522
ApoB48, mean (SD)	7.61 (3.23)	7.24 (3.34)	0.302
ApoB total, mean (SD)	96.89 (16.59)	94.00 (16.45)	0.251
TG-ChM/VLDLTG ratio, median (IQR)	1.08 (0.77–1.77)	1.46 (0.83–2.23)	0.099
VLDL/TG ratio, median (IQR)	0.19 (0.15–0.23)	0.20 (0–16-0.23)	0.822
TG/ApoB ratio, median (IQR)	5.54 (5.20–5.81)	5.50 (5.16–5.75)	0.304
NHDL-C/ApoB ratio, median (IQR)	0.49 (0.36–0.62)	0.50 (0.37–0.71)	0.466
Lipoprotein (a), median (IQR)	18.54 (11.90–36.77)	21.74 (12.88–41.90)	0.434
Preprandial carbohydrate profile			
Baseline glycemia (mg/dL), median (IQR)	78.00 (73.00–87.00)	81.00 (73.00–91.00)	0.181
Homocysteine, median (IQR)	13.70 (11.22–15.67)	12.40 (10.80–15.20)	0.186
Postprandial lipid profile			
Total cholesterol (mg/dL), mean (SD)	197.38 (33.25)	192.16 (32.99)	0.356
LDL cholesterol (mg/dL), mean (SD)	112.63 (30.39)	103.57 (28.40)	0.073
HDL cholesterol (mg/dL), mean (SD)	58.90 (13.00)	61.98 (16.40)	0.250
Triglycerides (mg/dL), median (IQR)	134.10 (91.75–195.00)	130.00 (90.50–174.00)	0.572
Triglycerides >220 mg/dL, *n* (%)	14 (18.70)	8 (11.90)	0.269
ChMTG, median (IQR)	48.80 (32.25–110.80)	44.90 (25.32–79.00)	0.898
ChMchol, median (IQR)	16.35 (10.70–23.75)	10.90 (7.90–18.20)	0.174
VLDLTG, median (IQR)	21.15 (11.15–28.17)	17.40 (12.20–30.30)	0.764
VLDLchol, median (IQR)	4.90 (2.80–8.80)	5.90 (3.82–10.30)	0.221
ApoB48, mean (SD)	14.38 (5.63)	12.63 (4.33)	0.041
ApoB48 p75, *n* (%)	24 (32.00)	10 (14.90)	0.017
ApoB total, mean (SD)	91.91 (15.45)	87.82 (16.38)	0.589
PPLH *, *n* (%)	29 (38.70)	15 (22.40)	0.036
Postprandial carbohydrate profile			
Glycemia (mg/dL), median (IQR)	92.00 (82.00–101.00)	86.00 (79.00–97.50)	0.100
Increase in postprandial lipidemia			
Triglycerides (mg/dL), median (IQR)	48.40 (28.40–76.40)	44.20 (17.80–82.70)	0.630
ChMTG, median (IQR)	31.50 (16.12–48.02)	25.10 (10.77–44.20)	0.132
VLDLTG, median (IQR)	10.15 (0.37–16.77)	8.65 (3.17–19.15)	0.820
ApoB48, mean (SD)	6.82 (4.55)	5.39 (3.86)	0.043

* PPHL: postprandial hyperlipidemia (TG > 220 mg/dL or ApoB48 > p75). Abbreviations. LDL: low-density lipoprotein; HDL: high-density lipoprotein; TG: triglycerides; ChM: chylomicrons; chol: cholesterol; NHDL: non-HDL cholesterol; VLDL: very-low-density lipoprotein.

**Table 4:** Clinical characteristics, cIMT, and cytokine profile associated with postprandial hyperlipidemia in patients with rheumatoid arthritis.

Variable	RA with PPHL * n = 29	RA without PPHL *n* = 46	*p* Value
Epidemiological characteristics			
Age in years, mean (SD)	56.80 (11.68)	53.66 (11.27)	0.250
Female sex; *n* (%)	23 (79.30)	41 (89.10)	0.242
Smoking			0.048
Never, *n* (%)	9 (31.00)	25 (54.30)	
Ex-smoker, *n* (%)	20 (69.00)	21 (45.70)	
Comorbid conditions			
Arterial hypertension, *n* (%)	10 (34.50)	11 (23.90)	0.321
Diabetes mellitus, *n* (%)	2 (6.90)	1 (2.20)	0.309
Cardiovascular disease, *n* (%)	3 (10.30)	2 (4.30)	0.311
Anthropometric characteristics			
BMI (kg/m^2^), mean (SD)	29.12 (5.90)	26.72 (4.60)	0.053
Obesity (BMI ≥ 30), *n* (%)	11 (37.90)	6 (13.30)	0.014
Waist circumference, (cm), median (IQR)	99.00 (86.00–108.00)	89.00 (83.00–95.50)	0.066
Hip circumference (cm), median (IQR)	104.00 (100.50–111.50)	105.00 (98.50–110.00)	0.780
Waist–hip ratio, median (IQR)	0.89 (0.84–0.96)	0.84 (0.80–0.89)	0.007
MET-minutes, median (IQR)	400.00 (182.50–902.90)	520.00 (275.00–890.00)	0.379
Total MEDAS score, median (IQR)	9.00 (8.00–10.00)	9.00 (8.00–10.50)	0.532
Clinical–laboratory characteristics			
Duration of RA, months, mean (SD)	136.85 (65.63)	132.48 (75.24)	0.794
Diagnostic delay, months, median (IQR)	6.48 (5.72–15.00)	5.72 (5.64–8.11)	0.161
Erosions, *n* (%)	17 (58.60)	18 (40.00)	0.092
RF > 10, *n* (%)	25 (86.20)	37 (80.40)	0.520
ACPA > 20, *n* (%)	26 (89.70)	32 (69.60)	0.043
High-sensitivity CRP (mg/dL), median (IQR)	5.46 (2.77–9.88)	2.95 (1.22–4.57)	0.040
ESR (mm/h), median (IQR)	18.00 (11.00–30.50)	15.00 (6.75–23.00)	0.190
DAS28-ESR at cut-off, media (DS)	3.59 (1.37)	2.96 (1.21)	0.042
Remission–low activity, *n* (%)	13 (44.80)	32 (69.60)	0.033
Moderate–high activity, *n* (%)	16 (55.20)	14 (30.40)	0.033
HAQ, mean (SD)	1.19 (0.85)	0.91 (0.82)	0.210
csDMARD, *n* (%)	26 (89.70)	40 (87.00)	0.726
bDMARD, *n* (%)	14 (48.30)	22 (47.80)	0.970
Corticosteroids at cut-off, *n* (%)	10 (34.50)	14 (30.40)	0.714
Carotid ultrasound			
Pathologic cIMT > p90, mean (SD)	16 (55.20)	10 (21.70)	0.003
Maximum cIMT (mm), mean (SD)	0.78 (0.16)	0.72 (0.11)	0.045
Right cIMT (mm), mean (SD)	0.72 (0.17)	0.67 (0.11)	0.260
Left cIMT (mm), mean (SD)	0.73 (0.15)	0.68 (0.10)	0.121
Patients with atheromatous plaque, *n* (%)	13 (44.80)	10 (22.20)	0.040
Two-sided involvement, *n* (%)	5 (17.90)	1 (2.20)	0.018
≥2 plaques, *n* (%)	6 (20.70)	3 (6.70)	0.066
Cytokines			
GM-CSF, pg/mL, median (IQR)	9.16 (7.91–11.46)	7.91 (7.01–10.39)	0.255
IFN-γ, pg/mL, median (IQR)	1.28 (0.93–1.98)	1.34 (1.02–2.26)	0.490
IL-1ß, pg/mL, median (IQR)	0.40 (0.32–0.55)	0.40 (0.32–0.45)	0.777
IL-12p70, pg/mL, median (IQR)	0.15 (0.10–0.30)	0.13 (0.09–0.19)	0.216
IL-13, pg/mL, median (IQR)	2.03 (1.77–3.03)	2.19 (1.84–2.68)	0.470
IL-18, pg/mL, median (IQR)	21.65 (10.80–26.75)	13.29 (10.17–19.23)	0.039
IL-2, pg/mL, median (IQR)	2.04 (2.00–2.42)	2.32 (2.04–2.51)	0.561
IL-4, pg/mL, median (IQR)	2.24 (1.92–3.25)	2.24 (1.92–2.57)	0.946
IL-5, pg/mL, median (IQR)	2.21 (1.78–2.98)	2.45 (2.21–2.92)	0.372
IL-6, pg/mL, median (IQR)	0.86 (0.57–2.48)	0.74 (0.56–1.66)	0.301
TNF-α, pg/mL, median (IQR)	1.49 (0.98–5.16)	1.14 (0.98–1.68)	0.036
IL-10, pg/mL, median (IQR)	0.18 (0.13–0.55)	0.20 (0.13–0.33)	0.872
IL-17A, pg/mL, median (IQR)	0.86 (0.59–3.26)	0.96 (0.59–2.16)	0.241
IL-21, pg/mL, median (IQR)	14.68 (2.04–182.14)	10.02 (2.21–56.04)	0.263
IL-22, pg/mL, median (IQR)	10.21 (2.31–155.49)	11.83 (3.65–142.65)	0.421
IL-23, pg/mL, median (IQR)	0.42 (0.40–0.52)	0.40 (0.40–0.52)	0.325
IL-27, pg/mL, median (IQR)	6.28 (4.93–8.85)	6.12 (4.93–7.64)	0.261
IL-9, pg/mL, median (IQR)	0.15 (0.10–0.24)	0.13 (0.10–0.19)	0.219
IFN-α, pg/mL, median (IQR)	0.09 (0.08–0.12)	0.09 (0.08–0.11)	0.581
IL-1α, pg/mL, median (IQR)	2.79 (0.42–42.14)	2.36 (0.49–14.14)	0.396
IL-15, pg/mL, median (IQR)	2.80 (2.53–3.61)	2.91 (2.70–3.38)	0.456
IL-1RA, pg/mL, median (IQR)	92.89 (69.88–268.76)	92.08 (60.30–300.78)	0.756
IL-31, pg/mL, median (IQR)	0.91 (0.78–1.06)	0.91 (0.78–0.98)	0.401
IL-7, pg/mL, median (IQR)	0.93 (0.60–1.69)	1.23 (0.76–1.73)	0.247
TNF-ß, pg/mL, median (IQR)	0.11 (0.10–0.13)	0.11 (0.10–0.14)	0.856

* PPHL: postprandial hyperlipidemia (TG > 220 mg/dL or ApoB48 > p75). Abbreviation: ACPA: anticyclic citrullinated peptide antibody; RF: rheumatoid factor; SD: standard deviation; MEDAS: Mediterranean Diet Adherence Screener (validated questionnaire); DAS28-ESR (28-joint disease activity score); CRP: C-reactive protein; ESR: erythrocyte sedimentation rate; GM-CSF: granulocyte macrophage-colony stimulating factor online; csDMARD: conventional synthetic disease-modifying antirheumatic drug; bDMARD: biologic DMARD; Il-6: interleukin 6. Anti-TNF: anti–tumor necrosis factor; TG: triglycerides.

**Table 5:** Logistic regression model for factors associated with postprandial hyperlipidemia in the whole group.

Dependent Variable	Predictor	OR	95% CI	*p* Value
Postprandial hyperlipidemia *				
	RA group	2.178	1.797–4.729	0.045
	Obesity (BMI ≥ 30)	2.674	1.109–6.451	0.039
	Baseline TG, mg/dL	1.019	1.006–1.031	0.013

* Triglycerides >220 mg/dL or ApoB48 >p75. Nagelkerke R2 = 0.213. Variables included in the equation: age, sex, smoking, carotid atheromatous plaque, TNF-α, high-sensitivity CRP, METs, obesity (BMI ≥ 30), Baseline TG (triglycerides), RA group (AR vs. controls). Abbreviations: TNF: tumor necrosis factor; TG: triglycerides: CRP: C-reactive protein; BMI: body mass index; RA: rheumatoid arthritis.

**Table 6:** Logistic regression model for factors associated with postprandial hyperlipidemia in patients with RA.

Dependent Variable	Predictor	OR	95% CI	*p* Value
Postprandial hyperlipidemia *				
	Carotid atheromatous plaque	4.693	1.095–12.118	0.037
	TNF-α, pg/mL	2.002	1.005–3.988	0.048
	High-sensitivity CRP, mg/dL	1.102	1.014–1.196	0.022
	Baseline TG, mg/dL	1.024	1.001–1.049	0.049

* Triglycerides > 220 mg/dL or ApoB48 > p75. Nagelkerke R2 = 0.516. Variables included in the equation: age, sex, smoking, carotid atheromatous plaque, TNF-α, high-sensitivity CRP, ACPA-positive, erosions, ACPA, METs, obesity (BMI ≥ 30), Baseline TG (triglycerides). Abbreviations: TNF: tumor necrosis factor; TG: triglycerides: CRP: C-reactive protein; BMI: body mass index; ACPA: anticyclic citrullinated peptide antibody.

## Data Availability

Data presented in this study are available on request from the corresponding author.
